# Assessment of the effects of skin microneedling as adjuvant therapy for facial melasma: a pilot study

**DOI:** 10.1186/s12895-017-0066-5

**Published:** 2017-11-28

**Authors:** Emerson V. A. Lima, Mariana Modesto D. A. Lima, Mauricio Pedreira Paixão, Hélio Amante Miot

**Affiliations:** 1 Santa Casa de Misericórdia, Praça Fleming, 35/1201 Jaqueira, Recife, PE 52050-180 Brazil; 2 UNIFESP, São Paulo, SP Brazil; 3 Unesp Medical School, Botucatu, SP Brazil; 4 Departamento de Dermatologia, SN, Campus da Unesp, Botucatu, SP 18618-000 Brazil

**Keywords:** Melasma, Treatment, Microneedling, Quality of life, Masi

## Abstract

**Background:**

Melasma is a common chronic and relapsing acquired dyschromia. Skin microneedling was reported resulting sustained long-term improvement of recalcitrant melasma, however, the exact mechanism that promotes this skin lightening is not known. This study aimed to investigate clinical and histologic alterations promoted by skin microneedling in facial melasma.

**Methods:**

Open pilot trial including six women with facial refractory melasma submitted to two sessions of microneedling (1.5 mm) each 30 days followed by daily triple combination and broad-spectrum sunscreen. Comparison of pretreatment (T0) and 15 days after last microneedling procedure (T45) was made by standardized pictures, skin colorimetry, MASI, MELASQoL and histological parameters (haematoxylin-eosin, picrosirius-red, periodic acid Schiff and Fontana-Masson staining).

**Results:**

The age of the subjects varied from 34 to 46 years-old, the phototypes were III and IV (Fitzpatrick), and age of melasma onset was 20 to 38 years. Improvement of melasma was perceived in all subjects. There was a significant reduction of MASI score (−70%), MELASQoL (−55%) and increase in L* (+13%) colorimetric value (*p* < 0.03). All cases evidenced epithelium thickening, decrease in melanin pigmentation and densification of upper dermis collagen (*p* = 0.03). Patients were followed by 6 months under broad-spectrum sunscreen and triple combination without relapse.

**Conclusion:**

In addition to classic treatment (broad-spectrum sunscreen and triple combination), skin microneedling promoted clinical and histological improvement of refractory facial melasma.

## Background

Melasma is a chronic and relapsing acquired dyschromia due to an increased epidermal-melanin unit activity that affects sun-exposed areas mainly in women throughout the reproductive years [1, 2].

Due to its high prevalence, the involvement of visible photoexposed areas - such as the face, in patients at a competitive age, and the relative resistance to treatment, melasma inflicts major impact on quality of life [3–5].

Its pathogenesis is not fully understood, nevertheless there is evidence that melanogenesis in melasma differ from tanning and post-inflammatory hyperpigmentation as well as there is an involvement of the whole epidermal melanin unit in the process (not just hypertrophic melanocytes), mastocytes, fibroblast and endothelium derived cytokines, as well as there are upper dermal abnormalities different from other acquired pigmentary disorders [6–8].

Skin microneedling, or percutaneous collagen induction by needles, is a minimally invasive procedure that uses short fine needles to puncture the skin and stimulates fibroblast proliferation, release of growth factors and collagen production [9–11]. Long-term improvement of recalcitrant melasma after microneedling was reported in one case series, however, the exact mechanism that promotes skin lightening is not known [12, 13].

This study has investigated clinical and histologic alterations promoted by skin microneedling in facial melasma.

## Methods

We performed an open pilot trial including women with facial refractory facial melasma, without specific treatment besides sunscreen for the last 30 days.

Refractory melasma was considered those with more than 5 years of evolution and relapsing to more than three attempting to treatment, including triple combination (hydroquinone, fluocinolone and tretinoin).

After consent, they were submitted to two sessions of microneedling (Dr. Roller™, 1.5 mm) each 30 days (T0 and T30), followed (at the next day) by daily triple combination (Tri-Luma, Galderma) application and broad-spectrum sunscreen (Anthelios Airlicium SPF 70 com cor, La Roche Posay), according to Lima protocol [13].

Standardised pictures, colorimetry (LED quasi-L*a*b*), skin biopsies (3 mm punch), MASI scores (range 0–48) and MelasQoL-PB questionnaire were taken at the inclusion visit (T0) and T45 [14–16]. The paraffin-embedded skin specimens were processed by haematoxylin-eosin, picrosirius-red, periodic acid Schiff and Fontana-Masson staining.

The study was performed at Santa Casa de Misericórdia (Recife-PE, Brazil), from October to November 2016, and was approved by institutional board review (Comitê de Ética em Pesquisa da Faculdade de Medicina de Botucatu-Unesp).

Variables were compared between T0 and T45 by paired Student’s t or Wilcoxon test if normality was not indicated by Shapiro-Wilk procedure [17].

Data was analysed at IBM-SPSS 24 and significance was set as two-sided *p* < 0.05 [18].

Sample size was calculated to detect at least 30% reduction of values of MASI scores between the visits, with an effect size (mean / standard deviation) of 1.1, alpha error of 0.05 and 80% of power [19].

## Results

The age of the subjects varied from 34 to 46 years-old, Fitzpatrick´ phototypes were III and IV, they reported 0 to 4 childbirths, daily time of direct sun exposure was 2 to 4 h, age of melasma onset was 20 to 38 years and MASI ranged from 29 to 46.

All participants have treated melasma previously with triple combination and others hydroquinone-free bleaching agents, with relapse.

**Fig. 1:**
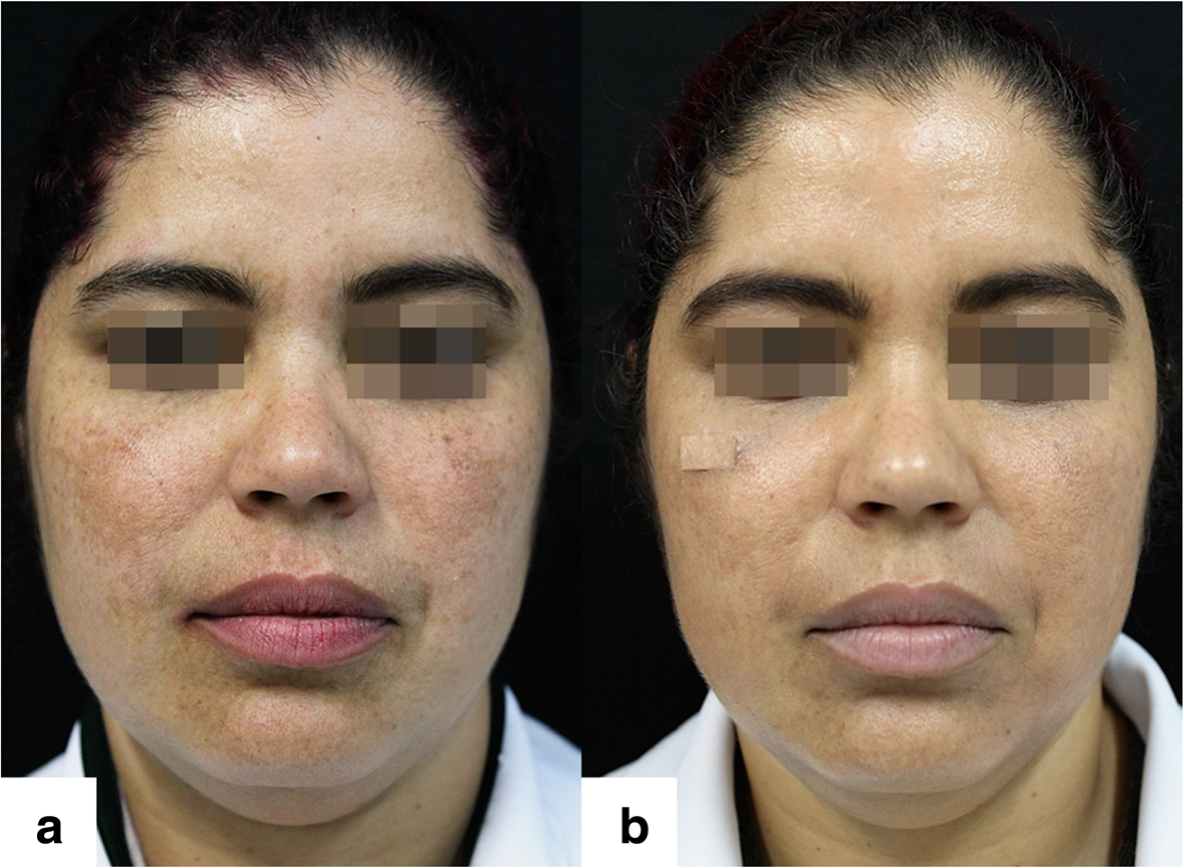
Facial melasma: **a** Pre-treatment (T0). **b** Post-treatment after two sessions of microneedling, triple combination, and broad-spectrum sunscreen (T45)

**Fig. 2:**
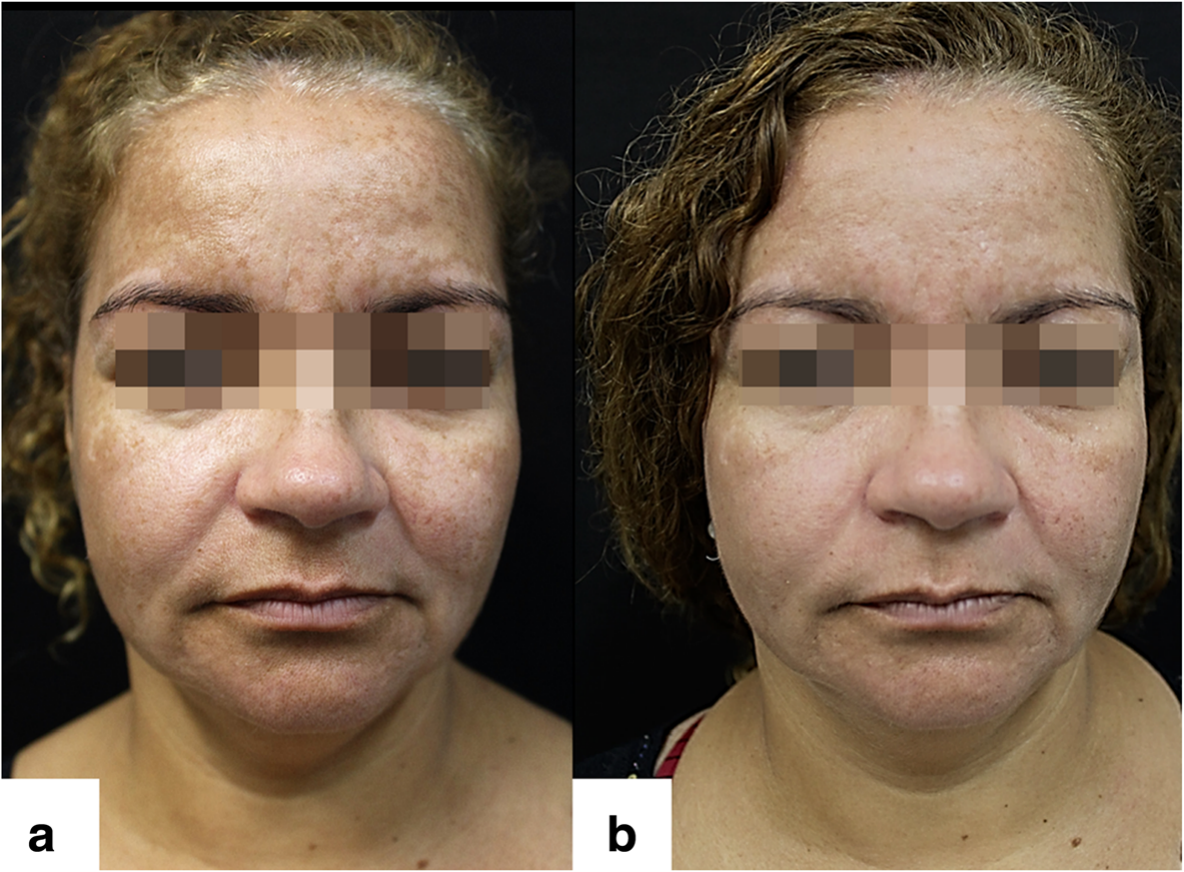
Facial melasma: **a** Pre-treatment (T0). **b** Post-treatment after two sessions of microneedling, triple combination, and broad-spectrum sunscreen (T45)

After two sessions of microneedling, improvement of melasma was perceived in all subjects (Figs. 1 and 2), in addition, there was a subjective report of overall facial skin smoothness and greater radiance by the participants.

Clinical, quality of life and colorimetric measures at T0 and T45 are presented at Table 1. A 70% mean decrease in MASI, 13% increase in luminance (L*) and 55% decrease in MELASQoL were noticeable.

**Table 1:** Clinical, quality of life and colorimetric measures of melasma before and after two sessions of microneedling (*n* = 6)

	Pre-treatment (T0)	Post-treatment (T45)	*p*-value
MASI	37.1 (8.2)	11.0 (2.9)	0.001
Colorimetry			
L*	43.5 (3.4)	49.2 (2.5)	0.015
a*	20.7 (6.6)	15.5 (4.3)	0.237
b*	32.0 (3.5)	28.2 (2.6)	0.040
ITA^o^	−11.5 (6.3)	−1.7 (4.4)	0.028
MELASQoL	70 (68–70)	32 (22–41)	0.027

*MASI* Melasma Severity Index, *ITA* ^o^ Individual Typology Angle, *MELASQoL* Melasma Quality of Life Scale

**Fig. 3:**
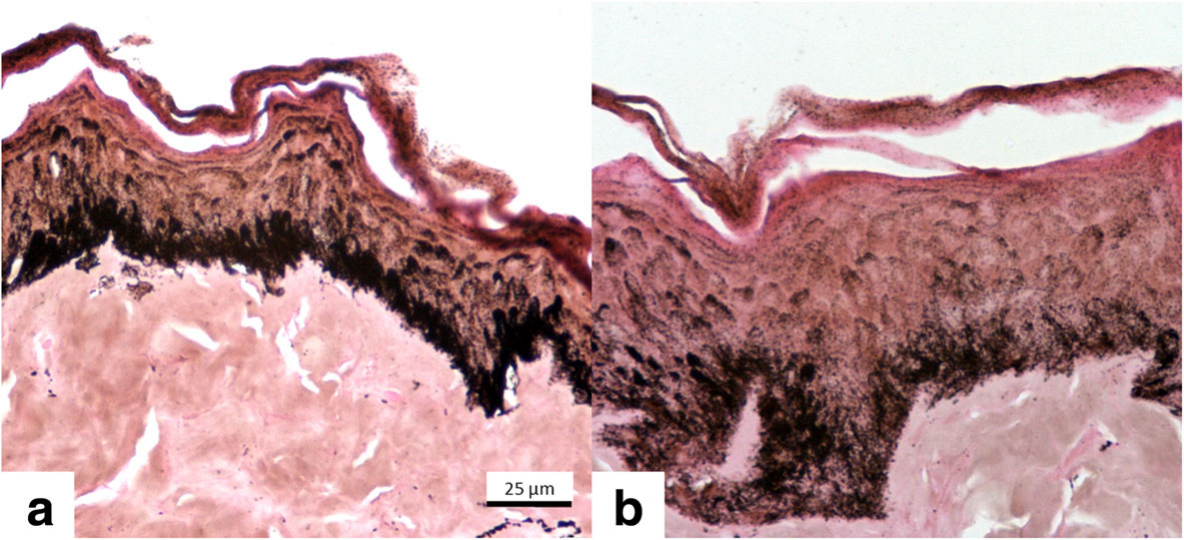
Fontana-Masson staining. **a** Pre-treatment (T0). **b** Post-treatment (T45), evidencing acanthosis and decrease in melanin density and the size of granules

**Fig. 4:**
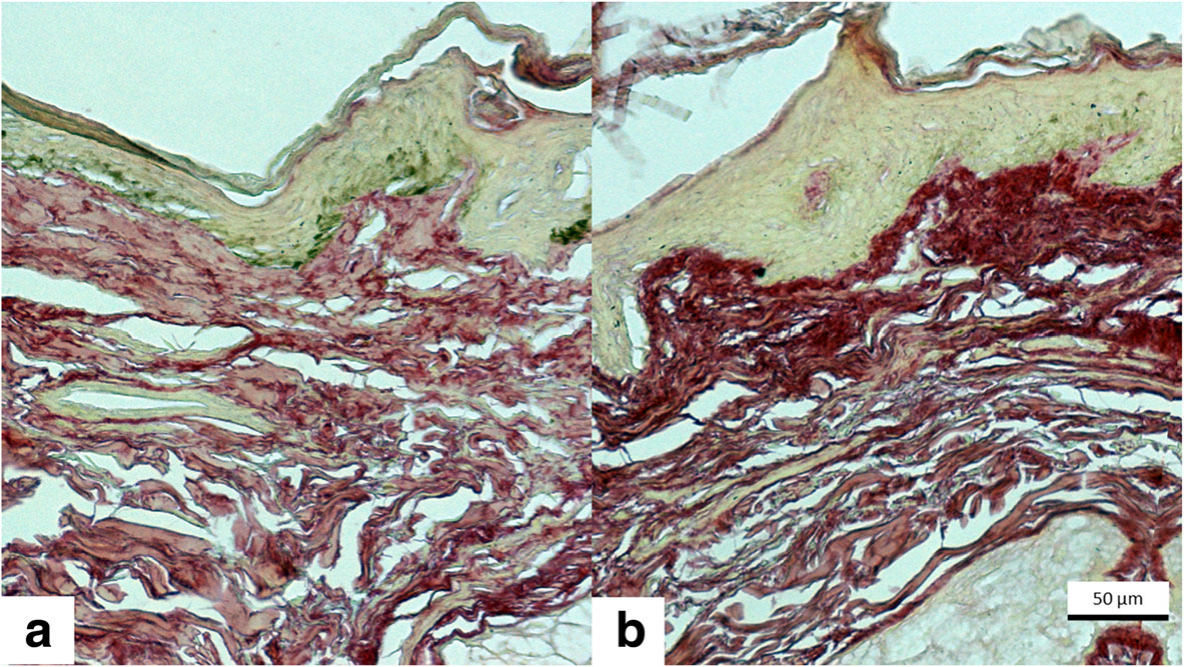
Picrosirius red staining. **a** Pre-treatment (T0). **b** Post-treatment (T45), evidencing upper dermal dense collagen bundles

**Fig. 5:**
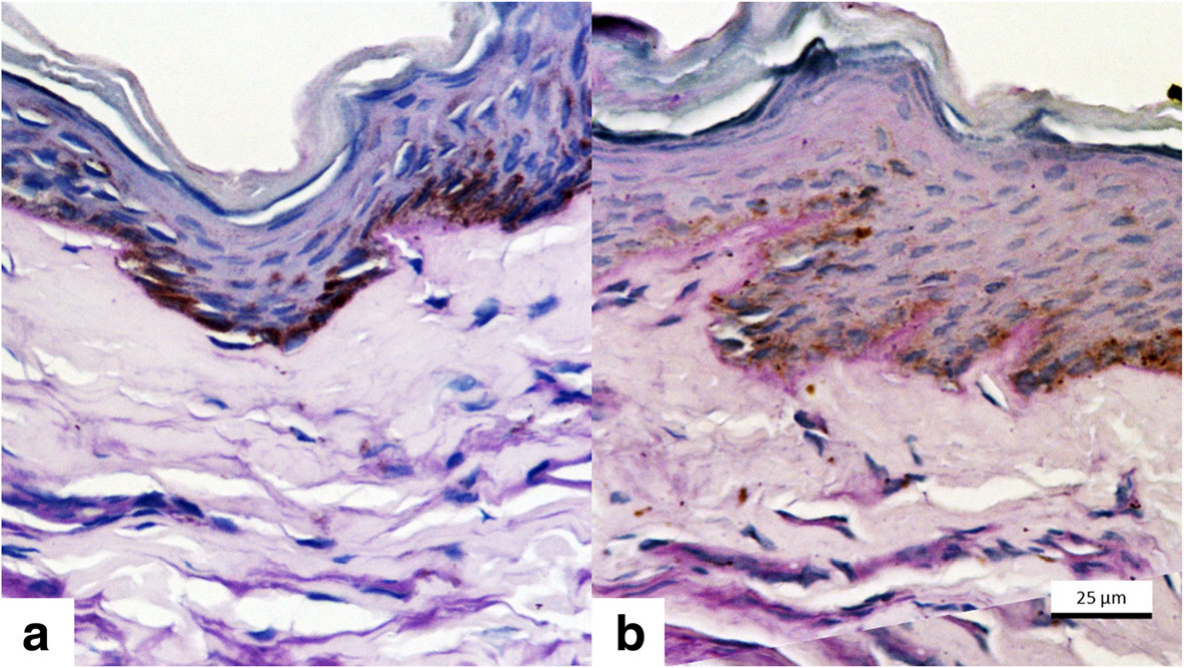
Periodic acid Schiff staining. **a** Pre-treatment (T0). **b** Post-treatment (T45), evidencing previous severe damaging followed by traces of restoration in the basement membrane zone

Histologically (Figs. 3 and 4), all cases evidenced epithelium thickening, decrease in epithelial melanin pigmentation and densification of upper dermis collagen (*p* = 0.03). Basement membrane (Fig. 5) was damaged in melasma, and there are traces of basement membrane restoration after the treatment.

Patients were followed by 6 months under broad-spectrum sunscreen and triple combination without relapse.

## Discussion

This is the first preliminary study that investigated clinical, quality of life, colorimetric and histological improvement in facial melasma with the addition of microneedling to the classic treatment.

In a historical comparison with a Brazilian population (*n* = 50) submitted to a regimen of broad-spectrum sunscreen and triple combination for 8 weeks, there was a mean MELASQoL reduction from 44.4 to 24.3 (45%) and a median MASI reduction from 13.1 to 3.2 (75%) [20]. Nevertheless, that sample had a less severe melasma than our patients, there were no reports of previous treatment relapses and the intervention lasted 25% more than our study (60 vs. 45 days).

Beyond clinical and quality of life improvement, epidermal melanin reduction, basement membrane restoration and increase in upper dermal collagen were evidenced. Basal membrane is damaged in melasma, as there are solar elastosis and collagen fragmentation what lead to the hypothesis of great activity of metalloproteinases [1–3, 9] and in upper dermis and decrease in type I collagen synthesis [8, 21–23].

Microneedling is classically indicated in the treatment of *striae distensae*, acne scars and photoaging, but its indications are widening among dermatologists [24, 25]. Some patients with acne scars perceived improvement in their melasma after microneedling, what motivated us to study a specific treatment regimen for them [13]. As it promote fibroblast proliferation and upper dermal collagenesis, microneedling can restore upper dermal and basal membrane damage in melasma, disfavouring the contact of melanocytes with dermal released melanogenic *stimuli* as endothelin, stem cell factor and hepatocyte growth factor [8, 22, 26, 27]. Additionally, a thickener epidermis can promote additional protection against UV damage.

In a previous histological study of triple association in melasma, the thickening of epidermis as well as upper dermal changes were not evidenced after 6 months of treatment [28]. This reinforces that the results we found in this preliminary study were induced by microneedling. Moreover, there is an increase in transepidermal drug delivery, for, at least, 72 h after the procedure. This can also increase the effect of triple association on the melanogenesis [29, 30].

Microneedling additional effects were suggested in a randomised controlled study with 60 patients comparing intradermal tranexamic acid versus its delivery by microneedling in facial melasma. There were respectively 36% and 44% improvement in MASI scores, moreover, 26% versus 41% of patients achieved 50% of MASI reduction [31]. Microneedling with vitamin C also resulted in a better clinical response followed Q-switched Nd:Yag for facial melasma, in a split-face trial with 16 patients [32].

Gentle dermabrasion with dental motor roller provided persistent clearance of melasma in 97% of 410 patients in a Thai case series [33]. The mechanism of dermabrasion related improvement of melasma was also not understood, however, as well as microneedling it promotes upper dermal neocollagenesis.

This study has potential limitations. It was performed in a single centre in the Northeast of Brazil (8°03′14″S and 34°52′52″W). Nevertheless, it is a sunny tropical city, what would disfavour the long-term remission observed at our follow up. The small sample size proposed in this pilot study also did not hamper we reach statistically significant results due to the consistency of the alterations induced by the treatment, as well as the main objective was to quantify clinical and histological alterations induced by microneedling in facial melasma. Finally, the addition of triple combination or the lack of a control group doesn’t allow to assess the effect of isolated microneedling in the treatment, nonetheless, these histological findings were not reported after triple combination and they make sense in the reversion of melasma pathogenetic issues. Moreover, microneedling can facilitate drug delivery of bleaching actives [28, 34, 35].

## Conclusions

In addition to classic treatment (broad-spectrum sunscreen and triple combination), skin microneedling promoted clinical and histological improvement of recalcitrant facial melasma. Further randomised controlled studies are warranted to investigate treatment regimens of microneedling in order to maximize its efficacy, as long-term maintenance of the results.

## Abbreviations

ITA^o^: Individual Typology Angle
MASI: Melasma Area Severity Index
MELASQoL: Melasma Quality of Life Scale
